# Identification of pro-angiogenic markers in blood vessels from stroked-affected brain tissue using laser-capture microdissection

**DOI:** 10.1186/1471-2164-10-113

**Published:** 2009-03-17

**Authors:** Mark Slevin, Jerzy Krupinski, Norma Rovira, Marta Turu, Ana Luque, Maribel Baldellou, Coral Sanfeliu, Nuria de Vera, Lina Badimon

**Affiliations:** 1 SBCHS, Manchester Metropolitan University, Manchester, UK; 2 Centro de Investigación Cardiovascular, CSIC-ICCC, Hospital de la Santa Creu i Sant Pau, Barcelona, Spain; 3 Hospital Universitari Mútua de Terrassa, Department of Neurology, Cerebrovascular Diseases Unit, Pl. Dr. Robert, 5, 08221 Terrassa, Barcelona, Spain; 4 Dept of Brain Ischemia and Neurodegeneration, IIBB, CSIC-IDIBAPS, Barcelona

## Abstract

**Background:**

Angiogenesis correlates with patient survival following acute ischaemic stroke, and survival of neurons is greatest in tissue undergoing angiogenesis. Angiogenesis is critical for the development of new microvessels and leads to re-formation of collateral circulation, reperfusion, enhanced neuronal survival and improved recovery.

**Results:**

Here, we have isolated active (CD105/Flt-1 positive) and inactive (CD105/Flt-1 minus (n=5) micro-vessel rich-regions from stroke-affected and contralateral tissue of patients using laser-capture micro-dissection. Areas were compared for pro- and anti-angiogenic gene expression using targeted TaqMan microfluidity cards containing 46 genes and real-time PCR. Further analysis of key gene de-regulation was performed by immunohistochemistry to define localization and expression patterns of identified markers and de novo synthesis by human brain microvessel endothelial cells (HBMEC) was examined following oxygen-glucose deprivation (OGD). Our data revealed that seven pro-angiogenic genes were notably up-regulated in CD105 positive microvessel rich regions. These were, beta-catenin, neural cell adhesion molecule (NRCAM), matrix metalloproteinase-2 (MMP-2), tissue inhibitor of matrix metalloproteinase-1 (TIMP-1), hepatocyte growth factor-alpha (HGF-alpha), monocyte chemottractant protein-1 (MCP-1) and and Tie-2 as well as c-kit. Immunohistochemistry demonstrated strong staining of MMP-2, HGF-alpha, MCP-1 and Tie-2 in stroke-associated regions of active remodeling in association with CD105 positive staining. In vitro, OGD stimulated production of Tie-2, MCP-1 and MMP-2 in HBMEC, demonstrated a de novo response to hypoxia.

**Conclusion:**

In this work we have identified concurrent activation of key angiogenic molecules associated with endothelial cell migration, differentiation and tube-formation, vessel stabilization and stem cell homing mechanisms in areas of revascularization.  Therapeutic stimulation of these processes in all areas of damaged tissue might improve morbidity and mortality from stroke.

## Background

Stroke is a leading cause of death and disability in the Western world. Neuronal survival in peri-infarcted regions determines the extent of patient recovery [1]. Patients with a higher density of blood vessels have reduced morbidity and mortality [2]. Restoration of cerebral microvascular circulation following angiogenesis/revascularization in peri-infarcted regions may salvage tissue, enhance neuronal survival and enhance functional recovery after stroke [3]. Following rat middle cerebral artery occlusion (MCAO), new blood vessels initiated through vascular buds, formed regular connections with intact microvessels within one week of ischaemia, with patterns similar to those in normal brain [4]. In disease situations, abnormally behaving cells are surrounded by heterogeneous tissue elements, and the areas of interest/diseased cells may constitute less than 5% of the volume of a sample. Conventional technology have employed microarrays to identify general changes in gene and protein regulation in biopsies from normal and abnormal regions of atherosclerotic plaques [5,3,6] and stroke tissue [7,8], but have failed to discover cell-specific changes, and in particular, those associated with angiogenesis. We hypothesise that within active regions of remodelling, angiogenic and non-angiogenic areas may co-exist. Laser-capture microdissection (LCM) can be used to isolate microvessels in evolving lesions. When combined with the latest RNA microscale extraction and analysis technology, this provides a powerful and sensitive tool for identification of genetic changes associated with blood vessel activation.

CD105 is the best known marker of active endothelial cells (EC) in diseased angiogenic tissues and is of prognostic value and a potential target for anti-angiogenic therapy in a variety of solid tumours [9-11]. CD105 is expressed by active EC making it the perfect target for identification of regions of tissue remodelling after stroke. The expression of CD105 in brain after stroke has not been studied, however, CD105 expression was induced by hypoxia in murine brain microvascular EC via mitogen activated protein kinase (MAPK) pathways [12] suggesting it is also a marker of active neovessel formation in ischaemic tissues. Using CD105 and Flt-1 as discriminating markers of microvessel activation, we aimed to identify the molecular fingerprint responsible for neovessel activation and revascularization following stroke.

## Results

**Figure 1:**
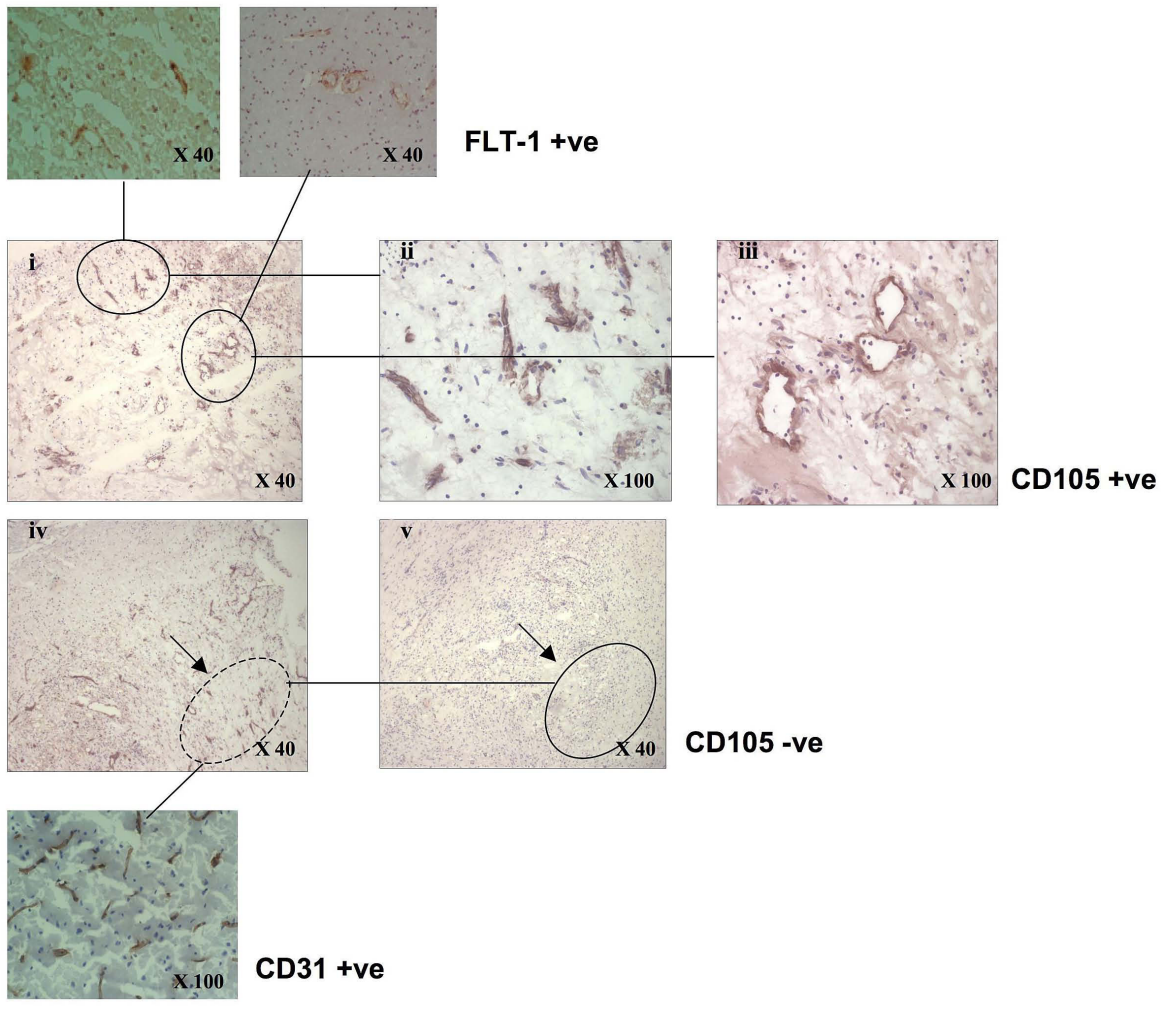
**Photomicrograph showing CD105-positive microvessels in histological areas chosen for laser-capture microvessels in peri-infarcted brain tissue (i-iii)**. CD105-positive clusters of blood vessels (inserts-top show the vessels were also Flt-1-positive. (iv) CD31-positive area (circled; insert) and (v) this area stained negative for CD105 (circle).

**Figure 2:**
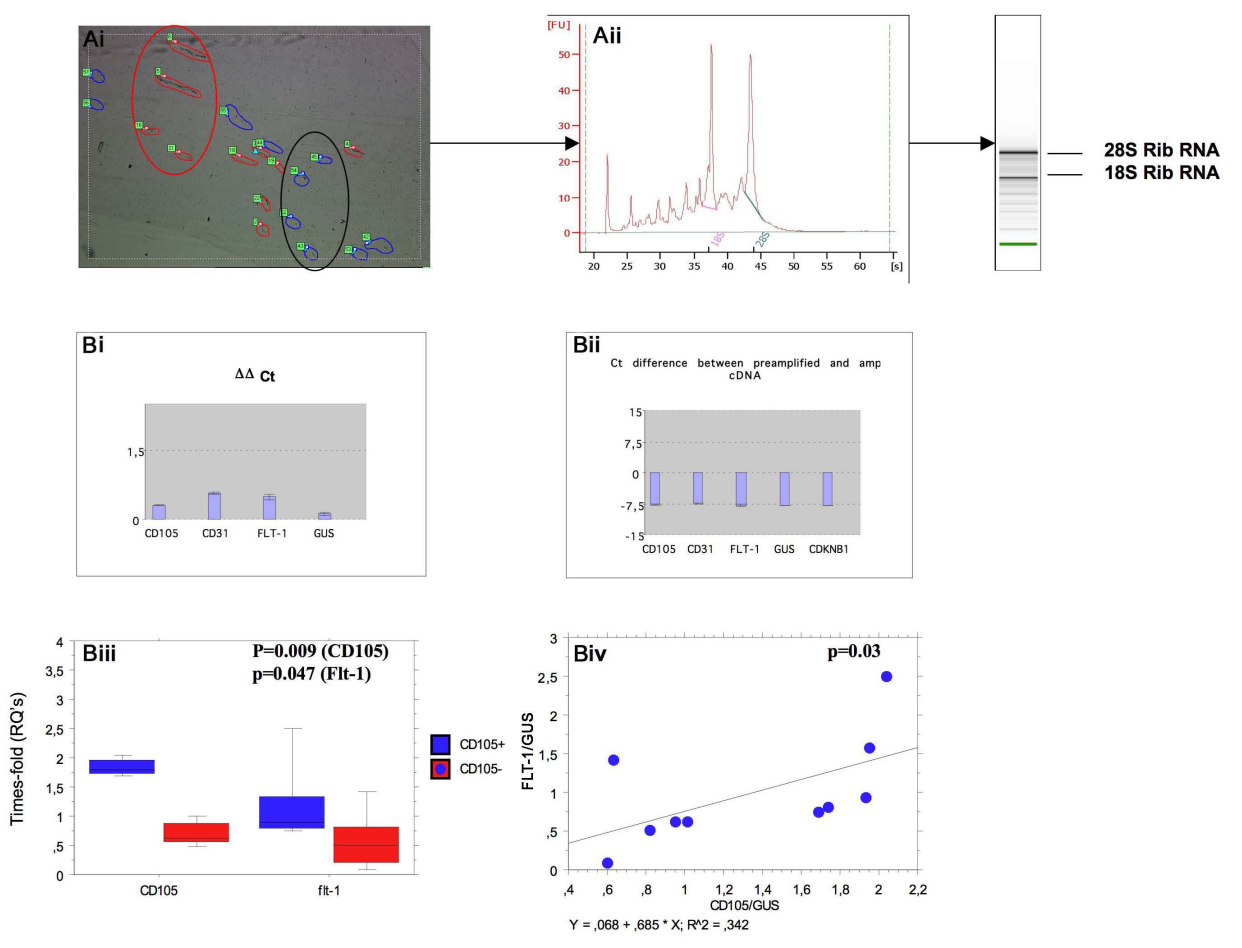
**A, (i) Area with discreet region of CD105-positive (red; circle) and CD105-negative (blue: circle) vessels captured with the laser**. (ii) RNA extraction from multiple combined laser cut sections showing good RIN and concentration. B, (i-ii) Shows non-significant differences in ΔCts between pre-amplified genes indicating no bias in gene amplification. (iii) Significant increases in gene expression of CD105 and Flt-1 in the positively selected samples indicated the sampling/IHC was accurate. (iv) Significant correlation between CD105 and Flt-1 expression in all the tested samples indicated reliability of the markers to discriminate between active and inactive vessels.

Areas rich in CD105-positive or CD31-positive/CD105-negative vessels were chosen for laser-capture as shown in (Figure 1). The cDNA obtained from 1 ng of total RNA was pre-amplified using the TaqMan Applied Biosystems PreAmp Master Mix Kit (Figure 2Ai–ii).

### Pre-amplification of RNA demonstrated equality of multiplication of key genes

Pre-amplification analysis demonstrated that Flt-1, CD105 and CD31 amplified to a similar extent and within acceptable limits with ΔΔCt lower than the cut off point of 1.5 and no significant differences between the Cts of any of the post-amplified genes (Figure 2Ci and 2Cii). Equality of gene amplification was tested using control (GUS), and CD31, CD105 and Flt-1.

### Selected immuno-positive CD105-positive tissue samples were enriched with CD105 and Flt-1 gene expression

Confirmation was made that the samples were CD31/CD105/Flt-1 enriched using TaqMan real-time PCR. Micro-areas chosen by laser-capture on the basis of CD105/Flt-1 IHC demonstrated significant increase in their gene expression (Figure 2Ciii; Mann Whitney U test; p = 0.009 and p = 0.047 respectively) and a strong correlation was also seen between CD105/GUS and Flt-1/GUS in all samples indicating accurate sampling of angiogenic and non-angiogenic regions (Spearman rank p = 0.03: Figure 2Civ). No significant difference in expression of CD31 in paired samples was seen (data not shown).

### TaqMan microfluidity card comparison of samples revealed significant differences in expression of key angiogenic genes correlating with CD105 and Flt-1 gene expression

**Figure 3:**
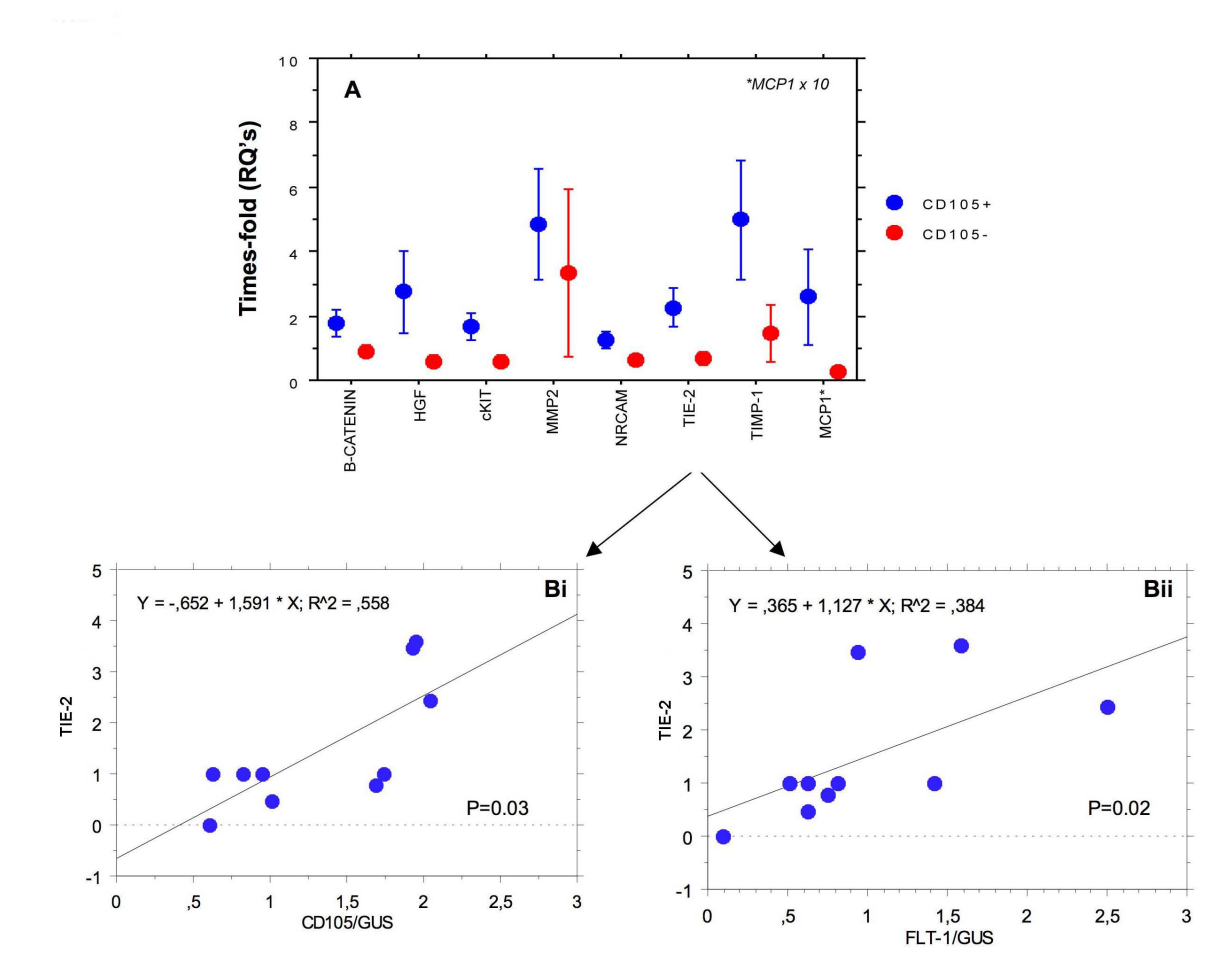
**A, shows relative expression of de-regulated angiogenic genes in CD105/Flt-1 positive (red) and negative (blue) samples**. Bi and ii, shows regression analysis and significant correlation (Spearman rank) between angiogenic gene expression and the markers CD105 (i) and Flt-1 (ii) for Tie-2, as an example. Other genes produced similar results.

Of the 47 genes tested, 7 pro-angiogenic genes were significantly up-regulated in CD105/Flt-1 positive vessel containing regions (c-kit, MCP-1, β-catenin, Tie-2, MMP-2, NRCAM and TIMP-1; Figure 3A and Bi and ii; Tie-2 shown) and a further one with RQ increased to > than 0.5 in 4/5 samples (HGF-α). None of the anti-angiogenic genes present in the arrays was significantly modified.

### Immunohistochemistry confirmed expression of HGF-α, MCP1, MMP-2 and Tie-2 in microvessels from stroked brain regions

**Figure 4:**
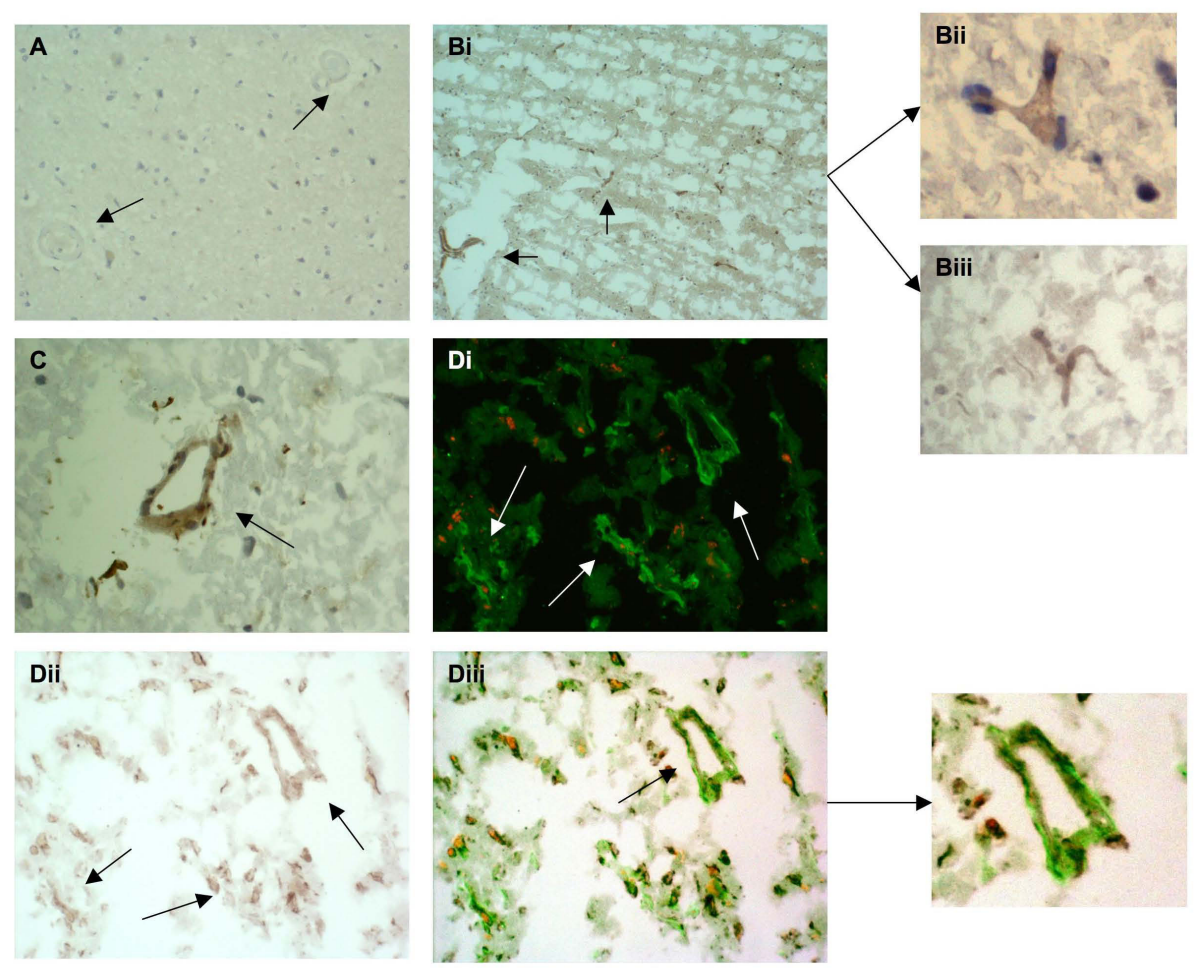
**HGF-α expression in stroke tissue: A, tissue from the contralateral hemisphere showed no observable staining (negatively stained blood vessels are marked with arrows; × 40)**. B, stroked brain tissue showing many small microvessels stained positive for HGF-α (i; × 40) and (ii and iii) at higher magnification (× 100). C, shows a medium sized microvessel strongly staining for HGF-α in peri-infarcted tissue (× 100) and Di-iii, double immunoflourescence showing co-localization of HGF-α (red) and CD105 (green) in peri-infarcted stroke tissue (× 100; sections from I0615P were used).

**Figure 5:**
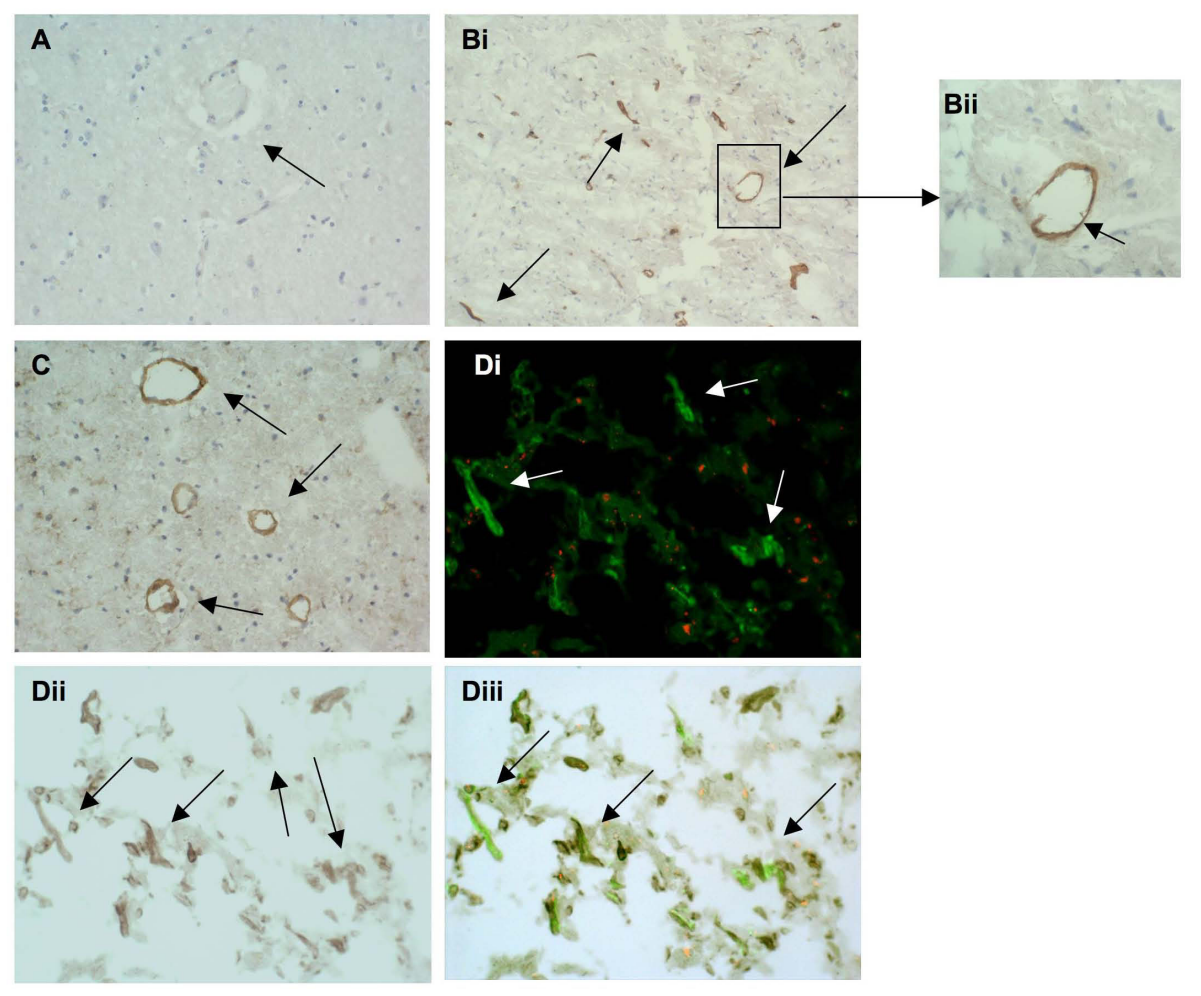
**A, tissue from the normal looking contralateral hemisphere showed no observable staining of Tie-2 (arrows; × 40)**. B, stroked brain tissue showing many microvessels strongly staining positive for Tie-2 (i; × 40) and (ii and iii) at higher magnification (× 100; arrows). C, small microvessels from the peri-infarcted zone positive for Tie-2 (× 100). Di-iii, double immunoflourescence showing co-localization of Tie-2 (red) and CD105 (green) in peri-infarcted stroke tissue (× 100; sections from I06232 were used).

**Figure 6:**
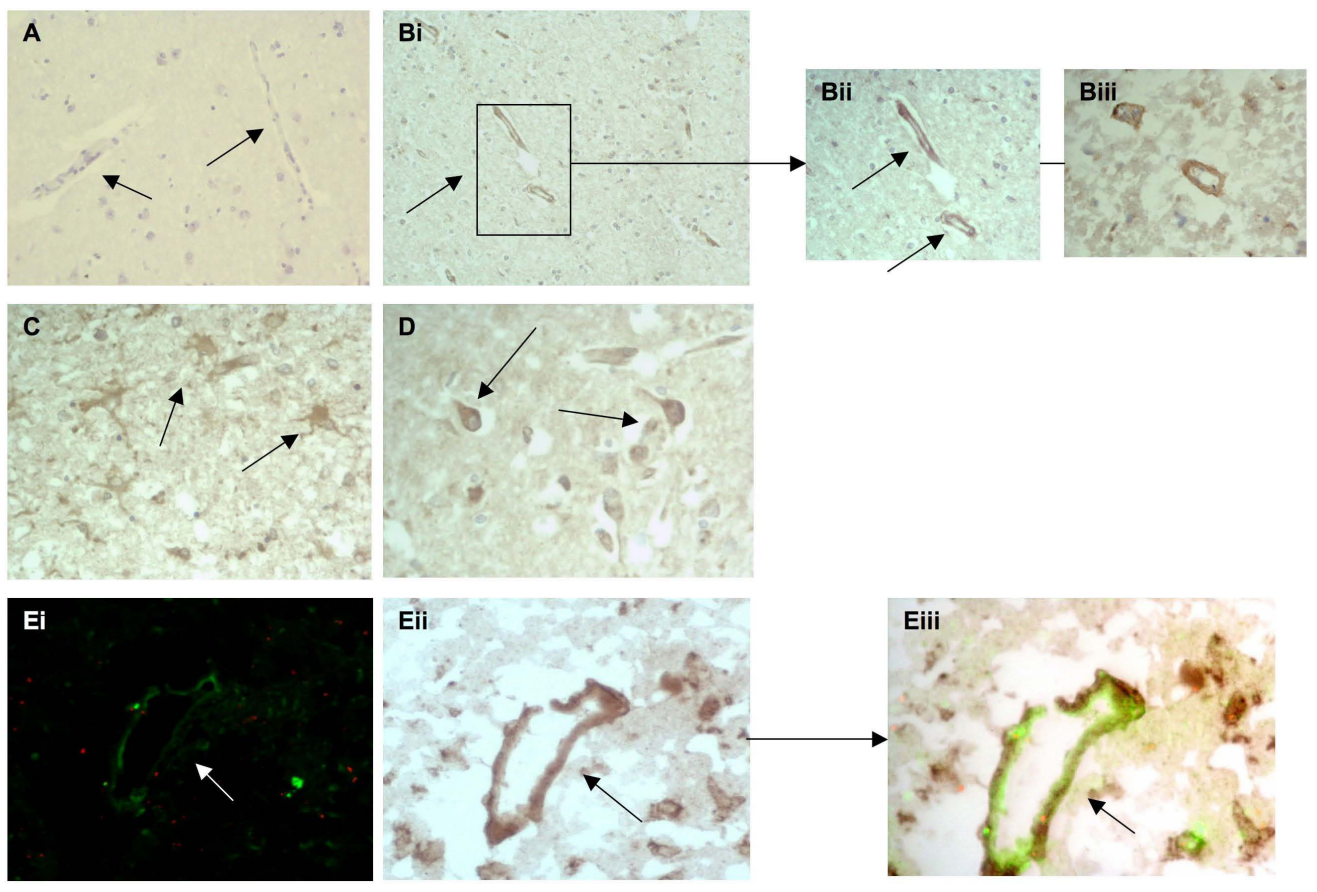
**MMP-2 was expressed strongly in stroked regions: A, tissue from the contralateral hemisphere showed no observable staining (negatively stained blood vessels are marked with arrows; × 40)**. B, peri-infarcted stroked brain tissue showing microvessels stained positive for MMP-2 (I; × 40; arrows) and (ii) at higher magnification (× 100) and (iii) in stroke/infarcted tissue (× 100; arrows). C, shows positive staining of cells with the morphological appearance of astrocytes/glia staining for MMP-2 in infarcted tissue (× 100; arrows) and D, cytoplasmic staining of neurones in the same region (× 100; arrows). Ei-iii, double immunoflourescence showing co-localization of MMP-2 (red) and CD105 (green) in peri-infarcted stroke tissue (× 100; arrow; sections from I0715P were used).

**Figure 7:**
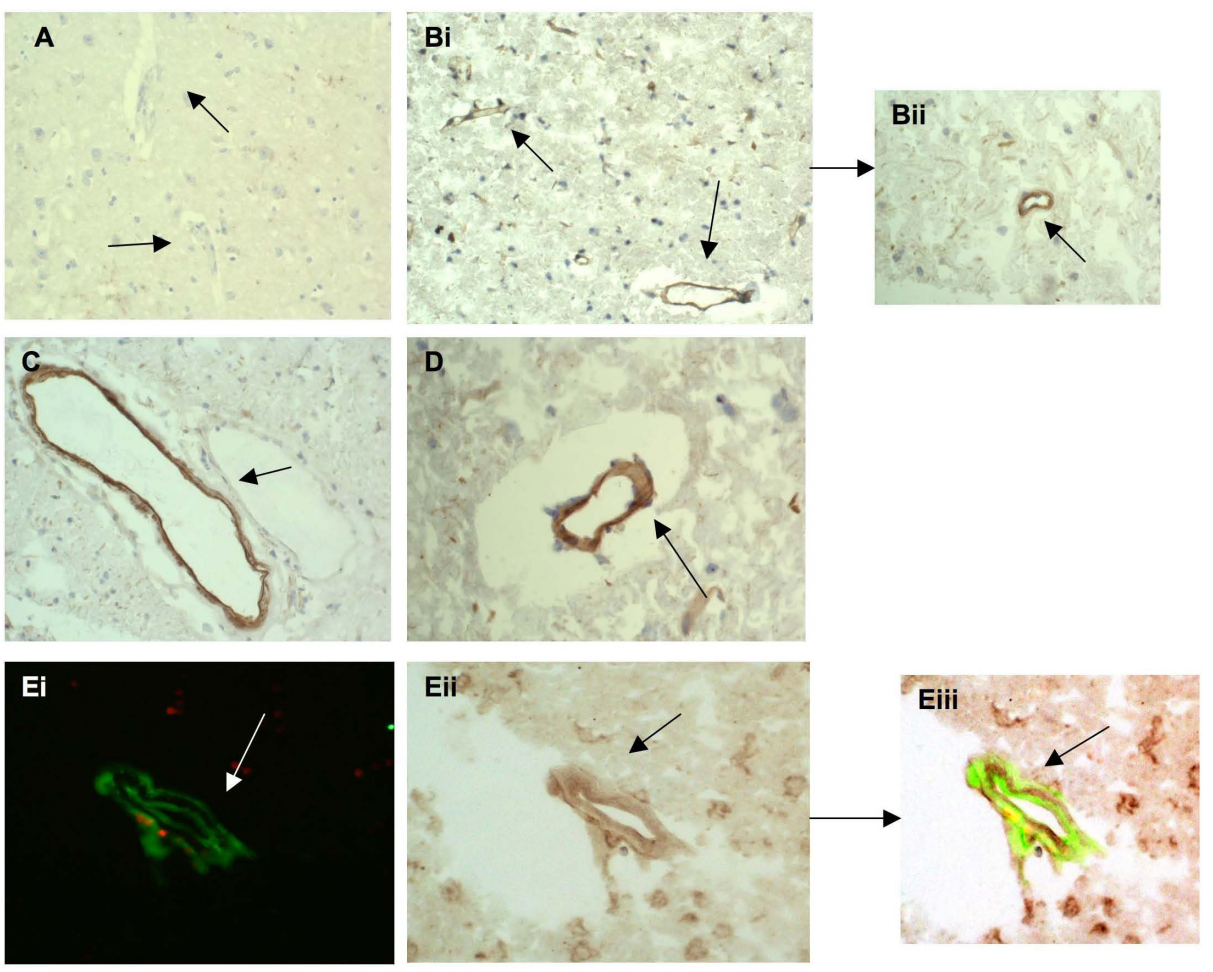
**A, tissue from the contralateral hemisphere showed no observable staining of MMP-2 (arrows; × 40)**. Bi and ii, stroked brain tissue showing many small microvessels stained positive for MCP-1 (× 40; arrows) C and D, show medium sized microvessels strongly staining for MCP-1; in C the arrow points to a positive vessel next to a negatively stained vessel in the peri-infarcted region (× 40; arrow) and in D, medium sized positively stained vessel in the infarcted region (× 100; arrow). Ei-iii, double immunoflourescence showing co-localization of MCP-1 (red) and CD105 (green) in peri-infarcted stroke tissue (× 100; sections from I06232 were used).

**HGF-α **was expressed strongly in microvessels of varying size from all active stroke regions after stroke (Figure 4A–C; Bi–iii). Old infarcted regions with dead cells were not stained. In contrast, there was no observable expression of HGF-α in normal looking blood vessels from the contralateral region. Glia and neurons were-unstained however, occasional inflammatory cells were positively stained in stroked regions. Many of the HGF-α-positive vessels, particularly those with malformed/impatent morphology stained CD105 positive suggesting cellular activation or angiogenesis was occurring in these areas (Figure 3D). **Tie-2 **showed a similar pattern with strong staining in small and medium sized, blood vessels from peri-infarcted and infarcted regions (Figure 5A–D; Bii shows higher powered micrographs; × 100). Again, there with no observable staining in sections from normal looking contralateral tissue (A; arrows). D; Tie-2 also co-localised in CD105-positive immature neo-vessels suggesting an association with angiogenesis. Tie-2 expression was specific for blood vessels with no staining of glia or inflammatory cells; however occasional dying neurons from the infarcted core had weak cytoplamic staining. **MMP-2 **was strongly expressed in both relative mature and immature microvessels from stroked regions, particularly in penumbral regions undergoing active remodeling (Figure 6Bi–iii and 6E, showing co-localization with CD105) and also in cells with the morphological appearance of astrocytes (Figure 6C) and neurons (Figure 6D). Normal looking (contralateral tissue was unstained (Figure 6A, arrows). **MCP-1 **stained primarily CD105-positive microvessels in peri-infarcted regions with some positive vessels also in the infarcted zones (Figure 7Bi–iii and 7C–E). Normal looking tissue did not stain for MCP-1 (A; arrows point to blood vessels). Some glia and infiltrating inflammatory cells were positively stained for MCP-1. In all cases, old infarcted regions stained negatively for all the antibodies tested and the majority of staining was seen in active regions of remodeling and revascularization. A summary of the findings is given in Table 1.

**Table 1:** Expression of novel angiogenic proteins in active regions of stroked brain tissue

**HGF-α**							
Code					In stroke affected areas		
	Age	Sex	Survival after stroke (days)	Neurones	EC	Glia	Inflamm cells
I06-256	77	M	52	-	++++	-	+
I06-139	84	F	7	-	++++	-	+
I07-30	68	M	19	-	+++	-	-
A06-77	75	F	3	-	++	-	-
I07-15	83	M	7	-	+++	-	+
I06-232	75	M	10	-	++	-	-
							
**MMP-2**							
Code					In stroke affected areas		
	Age	Sex	Survival after stroke (days)	Neurones	EC	Glia	Inflamm cells

I06-256	77	M	52	+	+++	+	-
I06-139	84	F	7	++	++++	+++	-
I07-30	68	M	19	++	+++	++	-
A06-77	75	F	3	+	++	+	-
I07-15	83	M	7	++	++++	+++	-
I06-232	75	M	10	+	++	+	-
							
**MCP-1**							
Code					In stroke affected areas		
	Age	Sex	Survival after stroke (days)	Neurones	EC	Glia	Inflamm cells

I06-256	77	M	52	-	+++	-	+
I06-139	84	F	7	-	++++	-	++
I07-30	68	M	19	-	++	-	+
A06-77	75	F	3	-	+++	-	++
I07-15	83	M	7	-	++++	-	++-
I06-232	75	M	10	-	+	-	+-
							
**Tie-2**							
Code					In stroke affected areas		
	Age	Sex	Survival after stroke (days)	Neurones	EC	Glia	Inflamm cells

I06-256	77	M	52	+	+++	-	-
I06-139	84	F	7	-	+++	-	-
I07-30	68	M	19	++	+++	-	-
A06-77	75	F	3	-	++	-	-
I07-15	83	M	7	-	++	-	-
I06-232	75	M	10	+	++++	-	-

### Exposure of HBMEC to OGD in vitro resulted in up regulation of Tie-2, MCP-1 and MMP-2 protein

**Figure 8:**
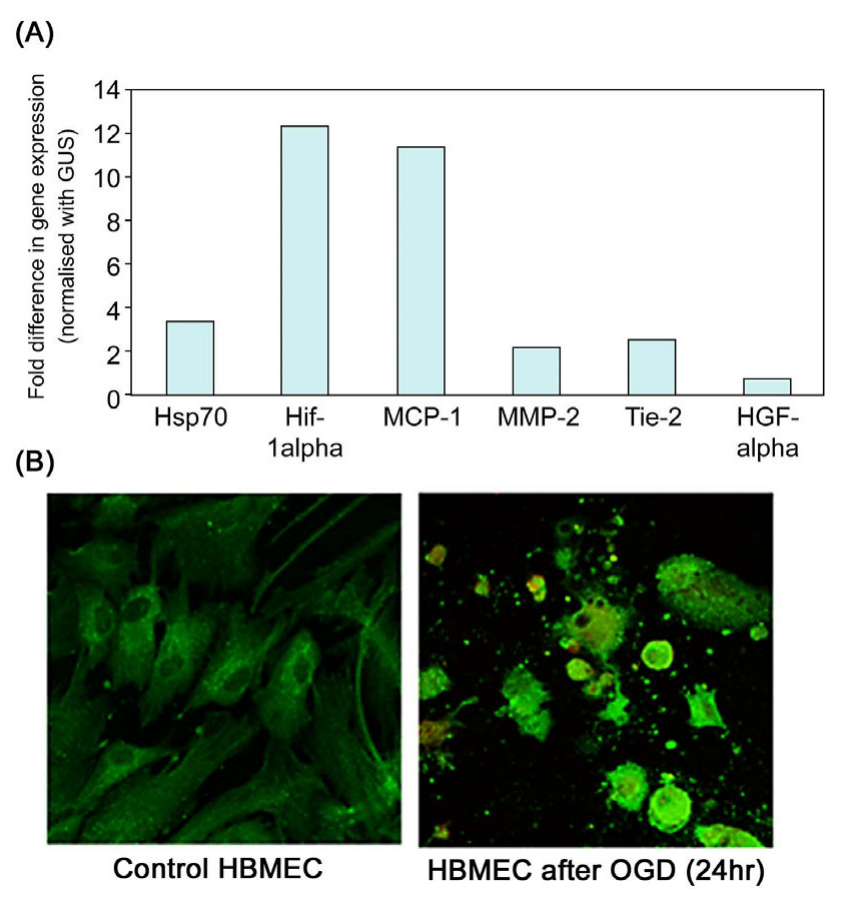
**A, Graph showing results from real-time RT-PCR with HBMEC exposed to 24 h OGD demonstrating a notable increase in HSP-70 and HIF-1α (control genes) and concomitantly an increase in expression of MMP-2, Tie-2 and MCP-1**. B, shows immunoflourescent staining of HBMEC for MMP-2. Control cells expressed MMP-2 weakly in peri-nuclear areas whilst HBMEC demonstrated a large increase in cytoplasmic expression following OGD (24 h) (× 100; MMP-2 stained green with FITC). All experiments were carried out twice in duplicate and representative pictures are shown.

When semi-confluent HBMEC were exposed to OGD for 24 h, increased gene expression of Tie-2 (2.5 fold), MCP-1 (11.4 fold) and MMP-2 (2.1 fold) were obtained by real-time PCR. Values were controlled using the house keeping gene GUS and a concomitant increase in expression of HIF-1α (12.4 fold) and Hsp70 (3.3 fold) demonstrated a strong response to the hypoxic environment (Figure 8A). All experiments were repeated twice and a representative example is shown. Immuno-staining showed that an increase in protein intensity was seen in the cytoplasm of PI-positive HBMEC following OGD (24 h) (Figure 8B, shows MMP-2).

## Discussion

Initiation and maintenance of angiogenesis in angiogenic diseases is a complex process requiring modulation of numerous pro- and anti-angiogenic molecules operating through complex intracellular signaling pathways. Identification of the key instigators of this process will help in defining future therapies for controlling vascularization. Here, for the first time to our knowledge, we have isolated micro-regions of angiogenic and quiescent microvessels from brain tissue of patients who died from acute ischaemic stroke and compared expression of the key angiogenic genes using real-time PCR and TaqMan microfluidity cards. From nanogram quantities of material, we have identified up-regulation of 7 genes with key roles in promotion of angiogenesis. Immunohistochemistry demonstrated a specific association of Tie-2, MCP-1, MMP-2 and HGF-α in peri-infarcted and infarcted CD105-positive blood vessels.

Laser-capture microdissection and RNA amplification technology has allowed the possibility to isolate and examine specific micro-sized cellular areas from heterogeneous tissue components. Previously, Hashimoto *et al*, [13], isolated vascular rich areas from synovial tissues and performed single-real-time PCR analysis on individual genes following RNA extraction. They demonstrated up-regulation of VEGF/VEGFR, HIF-1α, and inhibitor of differentiation-2 in blood vessels from inflamed regions of patients with rheumatoid arthritis. LCM was used to compare blood vessels from glioblastoma multiforme with those from vessels in normal brain tissue [14]. Pre-amplification of RNA followed by microarray analysis showed up-regulation of genes including insulin-like growth factor binding protein-7 and SPARC. Roy *et al*, [15], compared global gene expression between blood vessels isolated by laser-capture from normal skin and identified de-regulated genes from those within chronic wounded tissue, utilizing Ulex Europaeus Agglutinin (UEA1) which binds specifically to EC, to highlight the vessels. We also found that rapid staining with UEA1 in sterile water did not degrade the RNA whilst rapid immunostaining employing buffers did (our unpublished data). To discriminate between active and quiescent vessels, we labeled serial sections staining in groups of four using serial reference staining with anti-CD105, anti-CD31 and anti-Flt-1 antibodies for vessel identification. We confirmed that the areas chosen contained enriched markers of EC activation using RNA pre-amplification technology and house keeping controls. This showed that the relative amplification of genes was similar. One of the main aims of this work was to identify expression of angiogenic and anti-angiogenic factors produced in micro-regions of brain tissue in association with active micro-vessels. Therefore, in these experiments we carefully dissected concentrated areas of vessels including closely associated ECM encompassing any inflammatory components. In this way, we were able to gain an insight into the microenvironment to which the growing vessels were existing. Expression or synthesis of genes directly by the endothelial cells would feature as the main constituent due to their high relative concentration (identified by histology in all samples), however any component consisting of secreted factors from inflammatory infiltrates would also be seen giving us an overall view of the composition of these micro-hotspots.

Real-time PCR analysis of our microfluidity card data showed significant correlation in expression between de-regulated angiogenic genes and our markers of EC activation (CD105 and Flt-1) confirming the validity of our methodology. MCP-1 was originally identified as an important chemokine responsible for activation of macrophages and monocytes during inflammation but now is known to have a direct effect on EC mitogenesis in vitro and vessel formation in vivo [16,17]. The molecular mechanisms have not been dissected although Niu *et al*, demonstrated up-regulation of MCP-1-induced protein was necessary for VEGF and HIF-1α induction in HUVEC. We have shown that MCP-1 is strongly associated with active microvessels in peri-infarcted regions undergoing tissue remodeling after stroke.

We also showed a significant increase in Tie-2 expression in stroked regions. Both angiopoietin 1 and 2 can bind to the tyrosine kinase receptor Tie-2, which is responsible for vessel maturation and stability including facilitation of smooth muscle cell/pericyte attachment and therefore could be a key promoter of revascularization after stroke [18]. Simvastatin, used in treatment to lower cholesterol, is also angiogenic, and studies have shown that treatment with this drug following MCAO in a rat model, significantly increased EC capillary tube-formation dependent on induction of Tie-2 [19]. Studies using animal models have suggested that treatment with bone marrow stromal cells (MSC) after stroke, increases angiogenesis and tissue reperfusion in association with increased Tie-2 expression [20]. The same authors showed that capillary-like structure formation in mouse brain EC was increased in the presence of supernatant derived from MSC, whilst knock-down of Tie-2 inhibited this, suggesting an important role for Tie-2 in revascularization.

Hemorrhagic incident occurring after cerebral ischemia may be related to damage of the microvascular basal lamina of the brain, and can aggravate cerebral ischemia. This may be associated with up-regulation of MMPs and in particular, MMP-2 [21]. MMP-2 is up-regulated in EC exposed to inflammatory cytokines such as interleukin-1-beta and growth factors including nerve growth factor, where *in vivo*, it promotes capillary invasion and so is probably increased in active stroke regions undergoing remodelling [22,23]. Dong *et al*, [24], showed that resveratrol treatment 24 h–7 days after MCAO in mice increased MMP-2 and VEGF expression and concomitantly, the number of cortical microvessels as well as the neurological score, suggesting that MMP-2 has an important role in modulation of angiogenesis after stroke. This is in agreement with our data showing its association with CD105-positive microvessels in peri-infarcted regions.

We showed that expression of HGF-α was increased in the small neo-tubular vessels from peri-infarcted regions. Injection of human HGF gene with a hemagglutinising virus into rat CSF after MCAO, reduced neurological deficit within 24 hours of treatment and increased the number of microvessels in stroke-affected tissue [25]. The same authors showed that HGF-α gene transfer could significantly improve recovery of learning and memory concomitant with increased angiogenesis and neurite extension after stroke [26]. Rush *et al*, [27], demonstrated that addition of HGF-α to human brain microvessel EC, stimulated their migration through signalling pathways involving JNK, ERK and c-Src. This, together with the fact that HGF/c-met is also a chemoattractant for stroke-mobilized bone-marrow-derived stem cells [28], indicates that HGF could be a prime target for angiogenic therapy after stroke. Here, we also showed that HBMEC exposed to OGD demonstrated up-regulated HIF-1α and Hsp70 concomitant with MCP-1/MMP-2 and Tie-2 gene and protein expression suggesting at least some of the proteins may be produced by EC de novo after stroke.

For correct angiogenesis and maturation of vessels to take place, the time of expression and the number and type of angiogenic molecules effective in the vicinity of the developing microvessels, may be important, and this may vary dependent on the surrounding matrix. In our other studies (submitted elsewhere), we have shown using the same technology and identical TaqMan microarrays that the hypoxic environment associated with neovesel activation in carotid neointimal plaques induces expression of an overlapping, but certainly not identical group of angiogenic factors. In this case Tie-2 was also over-expressed as seen in this study, whilst the receptor for advanced glycation end-products (RAGE), angiopoietin-1 and Notch-3 were only increased in the plaque vascular bed. Therefore, the rate at which new microvessels are formed and/or are able to mature is probably governed by the number and concentration of relevant factors expressed. This may have important consequences in relation to attempts to induce therapeutic angiogenesis for the production of mature intimal vessels less prone to leakage and rupture, and the process may be site-specific. Future studies should aim to examine the effects of modulating these factors in terms of ratio and concentration with a view to optimising stable re-vascularization in vivo.

One of the ultimate clinical goals is to enhance and modulate the body's response to collateral blood vessel formation to maximise brain tissue reperfusion as rapidly as possible after stroke. Although we have not studied all the identified proteins in detail, pilot studies showed that β-catenin (pro-angiogenic and also mobilizes endothelial progenitor cells; [29] and TIMP-1 were also expressed strongly in stroke-affected microvessels, whilst c-Kit-positive cells were also present (data not shown).

Some of the limitations of this study include the small numbers of measured samples which has not allowed us to relate the findings directly to clinical information and stroke characteristics such as infarct size, extent of recovery, association with time of survival and survival. More extended detailed studies using larger patient cohorts could examine the information gained from this study and ascertain the importance of these proteins in mediating tissue reperfusion and any relationship with improvement in patient survival. Similarly, the data we have provided only allows us to infer what the overall effects of the presence of mixtures of both pro- and anti-angiogenic factors on microvessel formation, proliferation and maturation after stroke might be. Future experiments employing matrigel implant models may be able to determine the effects of introducing a mixture of factors such as those described in this study and examining in detail the formation structure and maturation of vessels over time.

## Conclusion

We hypothesise that combined processes including matrix degradation, direct activation of migration and proliferation of endothelial cells, attraction of bone marrow-derived stem cells and stabilization of new vessels with pericytes and smooth muscle cells are all vital components of this response, which is maintained many weeks after stroke in active regions of remodelling and therefore future therapeutic treatments might involve maximisation of all of these processes.

## Methods

### Patients

Tissue samples were collected within 4 hours of death from the refrigerated bodies of 6 patients who died 3–52 days after stroke following middle cerebral artery occlusion. Clinical details of these patients are supplied (Table 2). Samples were dissected into infarcted (identified with 2, 3, 5-triphenyltetrazolium chloride), peri-infarcted and normal looking unaffected tissue. Peri-infarcted region was defined as the area of tissue adjacent to the ischaemic core exhibiting stroke-related changes including neuronal apoptosis and angiogenesis and characterised by tissue oedema and discolouration, morphology of the neurons and maintenance of structural integrity. Tissue from the contralateral hemisphere served as a control. Tissue was frozen in liquid nitrogen, stored at -70°C and a sample processed for histology and stained with haematoxylin and eosin to determine tissue morphology. The usefulness of post-mortem samples in studies involving measurement of RNA and protein expression has been identified in previous studies [30]. Samples were obtained from the Cardiovascular Investigation Centre's Tissue Bank, St Pau Hospital, Barcelona, Spain, and ethical approval for the work was granted from the University Hospital of Bellvitge. Samples were dissected into 2 mm cross-sectional pieces and one portion reserved for laser-capture and microarray analysis and the other was fixed in 10% buffered saline prior to paraffin embedding.

**Table 2:** Clinical details of patients used in this study.

**Code**	**Size/Location of infarct**	**Age**	**Sex**	**Vascular risk factors**	**Immediate cause of death**	**Other disease**	**Stroke survival**	**Medications** **Statin/Anti-hypertensives**
I06-139	R MCA stroke atherothrombotic (TACI)*	84	M	Smoking, HTA, MI, CAD, Stroke, PVD	Large stroke	CRF, AD stage II B&B	5 days	Antiplatelets, Statins, Rasb
A06-77	L MCA stroke cardioembolic (TACI)	75	F	HTA, DM, CAD, stroke, valvulopathy,	Acute pulmonary edema	AD stage IIA B&B	3 days	Antiplatelets, Statins, Rasb
I06-232	L MCA atherothrombotic stroke (PACI)	75	M	Smoking habit, alcoholism, hypercholesterolemia	Respiratory infection	Alcoholic liver disease, chronic obstructive pulmonary disease	10 days	Statin
I06-256	R MCA stroke atherothrombotic (TACI)	77	M	Smoking habit, HTA, DM, atrial fibrillation	Large stroke, Respiratory infection	Colon adenocarcinoma	52 days	Antiplatelets, Statins, Rasb
I07-15	L MCA stroke atherothrombotic (TACI)	83	M	HTA, hypercholesterolemia, CAD	Respiratory infection	no	7 days	Statins, Rasb
I07-30	R MCA stroke atherothrombotic (TACI)	89	F	none	Acute pulonary edema, respiratory infection	no	21 days	none

**Abbreviations**: MCA, middle cerebral artery; HTA, hypertension; CRF, chronic renal faliure; DM, diabetes mellitus; CAD, coronary artery disease; AD, Alzheimer's disease; PVD, peripheral arterial disease; MI, myocardial infarction. B&B is Brack and Brack classification; Rasb, rennin-angiotensin blockers; *Oxfordshire classification; R, right side; L; left side TACI; Total anterior circulation infarct; PACI; Partial anterior circulation infarct.

### Immunohistochemistry

Frozen sections were labelled to identify EC using antibodies to CD31, and the specific markers of microvessel angiogenesis Flt-1 (VEGF receptor-1; [31] and CD105 [32] using standard ABC protocols, DAB colour development and haematoxylin counterstain. Negative controls replacing the primary antibody with PBS or non-immune serum demonstrated specificity. Dissected tissue areas were placed into categories of either; active content of microvessels with many Flt-1 and/or CD105 positive EC or inactive with few vessels expressing these markers. Subsequently, antibodies to proteins related to several of the novel, identified de-regulated genes from the TaqMan microarrays (MMP-2, Tie-2, MCP-1 and HGF-α) were used to determine the expression and localization within normal and stroked brain regions. Co-localization studies employed subsequent staining with anti-CD105-FITC (1:50).

### LCM

Serial 6 μm frozen sections were taken from microvessel-rich brain samples. Areas were scanned by labelling adjacent sections with CD105 and CD31 to estimate regions containing high concentrations of CD105 positive vessels (Figure 1) Sections were taken in groups of four. The first stained for CD105, the second prepared on a matrix (transparent ethylene-vinyl film) coated slide for laser-capture and stained only with haematoxylin prior to laser-capture (to reveal morphology), the third stained with anti-CD31 and the fourth anti-Flt-1. Photomicrographs of each stained section were taken and each area analysed by a pathologist. Areas rich in positive or negative microvessels were marked. LCM was performed on the middle section using a Robot Microbeam Laser Microscope (P.A.L.M Microlaser Technologies, Bernried, Germany). The laser diameter was set to 15 μm and small (<200 μM) CD105+/- microvessel rich areas were selected with close reference to the stained serial sections (Figure 2A). Captured areas were collected on the cap of a microcentrifuge tube and homogenized in RLT buffer (RNeasy micro-kit, Quiagen). If the laser did not succeed in releasing the tissue completely, the resultant marked area was carefully removed by an experienced dissection microscopist and placed directly into RLT buffer. At least 5 groups of sections were treated like this and the laser cut areas pooled to ensure sufficient numbers of cells were obtained for RNA analysis. Selected microvessels/cells were extracted and RNA purified using the QIAGEN RNeasy Micro kit (Hilden, Germany). The quantity and quality of RNA was assessed using a 2100 Bioanalyser (Agilent Technologies). The cDNA obtained from 1 ng of total RNA was pre-amplified using the TaqMan Applied Biosystems PreAmp Master Mix Kit and equality of gene amplification tested using control (GUS), and CD31, CD105 and Flt-1 (Figure 2Ci and 2Cii). Confirmation was made that the samples were CD31/CD105/Flt-1 enriched using TaqMan real-time PCR (Figure 2Ciii and 2Civ) showing correlation between CD105 expression and Flt-1 indicating both were associated with active samples (Spearman Rank; p = 0.025; Rho = 0.742).

### TaqMan arrays

A customised pre-configured 48 TaqMan Gene Expression assay (Applied Biosystems, CA, USA) in a 384-well format, spotted on a microfluidic card was used (2 replicates per assay; if the data generated (Cts) differed by greater than 0.35 SD the result was discarded). Real-time RT-PCR amplifications were run on an ABI Prism^® ^7900 Ht sequence Detection System (Applied Biosystems) with a TaqMan Low Density Array Upgrade. 5–22 ng of cDNA was combined with TaqMan universal master mix, and real-time PCR carried out according to the manufacturer's instructions. Our LDA contained 46 candidate markers derived from published data on wound recovery, tumour angiogenesis, angiogenesis in other diseases including stroke and diabetic retinopathy, and from our own and other microarray studies on atherosclerosis. [3]; Table 3. Paired control and CD105 positive samples were chosen for comparison to eliminate inter-plate variation and each pair had similar RNA integrity Number and the same loading cDNA concentration (see statistical analysis for further information).

**Table 3:** List of genes selected for use in the LDA.

Kallistatin	Angiopoietin
Transforming growth factor-beta	CD13

Platelet-derived growth factor	CD44

Vascular endothelial cell derived growth factor	c-Kit

Hepatocyte growth factor	Monocyte chemotactic protein 1

Epidermal growth factor	Tumour necrosis factor-alpha

Interleukin-8	Cyclooxygenase-2

Granular colony stimulating factor	Heparin-binding EGF-like growth factor

Hypoxia inducing factor-1	PR39

Thymosin β4	RANTES

Matrix metalloproteinases 1, 2, 3, 9	Thrombospondin 1

Receptor for advanced glycation end-products	T-cadherin

Integrin αV	DLL-4

Receptor for hyaluronan-mediated motility	C-reactive protein

Tie1-2	Toll-like receptor 2, and 4

Tissue inhibitor of metalloproteinase-1	Inhibitor of differentiation

Hyaluronidase-1	

### OGD

For OGD experiments, primary HBMEC were seeded on gelatin-coated glass coverslips. An aqueous solution of 1% porcine skin gelatine, type A (Sigma) was heated at 40°C up to complete dissolution, autoclaved and stored at 4°C until use. Coating was performed for 1 hour at 37°C, then gelatine was discarded and the coverslips washed with PBS. Experiments were performed in an anoxic chamber at 37°C. Cells in coverslips were maintained inside a Petri dish with medium without glucose in an atmosphere of N_2 _95%/CO_2 _5% for 24 h (pilot studies demonstrated at this time cells showed morphological evidence of stress, cytoplasmic shrinkage and propidium iodide uptake (PI) demonstrating DNA damage and apoptosis. Cultures were stained with propidium iodide (7.5 μg/ml) for 30 min to identify cell membrane damage, washed with PBS and fixed with 4% paraformaldehyde. Nuclei were then stained with bisbenzimide (5 μM) for 30 min. Gene expression was measured in duplicate samples using Real time PCR and nucleotide sequences obtained from TaqMan (identical to those used in the microarrays) using the method described above. Immunos-staining was carried out directly on the coverslips as described above and using FITC-conjugated secondary antibodies (1:100). All experiments were performed twice.

### Statistical analysis

cDNA samples were paired for TaqMan analysis to reduce inter-plate variation. Samples were chosen based on the RIN value and the amount of cDNA loaded. If RQ values between duplicates differed by >0.1 the sample was discarded. Differences in gene expression between individual pairs were considered if the RQ difference was ≥ 0.5. When 4 out of 5 samples showed differences statistical analysis was carried out. Using GUS as a house keeping control, non-parametric testing was carried out comparing expression between CD105 positive and negative samples (Mann Whitney U test), and Spearman Rank correlation of CD105 and Flt-1 expression with the genes of interest. Genes chosen for further analysis satisfied at least 2 of the following criteria: 1) 4/5 paired samples had RQ differences> 0.5; 2) Mann Whitney U test showed a significant difference between the two sets of 5 samples (p < 0.05) or 3) Spearman Rank correlation showed a significant correlation between CD105/Flt-1 expression and expression of the gene of interest.

## Authors' contributions

All the authors have read and approved the final manuscript. MS, LB and JK directed the work, MS carried out the laser-capture work and drafted the manuscript, MB and MT carried out the microarray work and statistical analysis, CS and NV did the OGD and NR and AL carried out the immunohistochemistry.
